# From invisibility to political power: Policy lessons from a decade of Brazil’s Social Forum for Infectious and Neglected Diseases

**DOI:** 10.1371/journal.pntd.0014336

**Published:** 2026-05-29

**Authors:** Eloan dos Santos Pinheiro, Carmem E. Leitão Araújo, Eleonora Schettini Martins Cunha, Aymée Medeiros da Rocha, Diogo Lopes Nunes Galvão, Eliana Amorim de Souza, Marina Pereira Cert-osé Alexandre Menezes da Silva, Alberto Novaes Ramos

**Affiliations:** 1 Former Director of Farmanguinhos, Oswaldo Cruz Foundation (Fiocruz), Rio de Janeiro, Rio de Janeiro, Brazil; 2 Department of Community Health, Faculty of Medicine, Federal University of Ceará, Fortaleza, Ceará, Brazil; 3 Department of Political Science, Federal University of Minas Gerais, Belo Horizonte, Minas Gerais, Brazil; 4 Research Coordination, Fundação NHR Brasil, Fortaleza, Ceará, Brazil; 5 Access Department, Neglected Diseases Programme, Drugs for Neglected Diseases initiative (DNDi), Rio de Janeiro, Rio de Janeiro, Brazil‌‌; 6 Multidisciplinary Institute in Health, Federal University of Bahia, Vitória da Conquista, Bahia, Brazil; 7 CUIDA Chagas Project, National Institute of Infectious Diseases, Oswaldo Cruz Foundation, Rio de Janeiro, Rio de Janeiro, Brazil; Institute of Development Studies, UNITED KINGDOM OF GREAT BRITAIN AND NORTHERN IRELAND

## Abstract

Neglected tropical diseases (NTDs) cluster among populations living in poverty and facing overlapping social, environmental, and political vulnerabilities. Brazil has one of the most significant NTD burdens in the Americas, yet affected populations have historically had limited influence on national health agendas. In 2016, amid democratic backsliding and fiscal austerity, civil society, researchers, and affected communities created the Brazilian Social Forum for Combating Infectious and Neglected Diseases (FSBEDIN) to strengthen political participation. Using documentary analysis of Forum letters (2016–2025), institutional records, and participant observation, we show how FSBEDIN evolved from a crisis-driven initiative into a recognized actor in Brazilian health governance. The Forum has linked disease-specific movements, expanded the presence of affected leaders in health councils and technical committees, supported leadership training, and helped catalyze the creation of a National Movement for Neglected Diseases. It also advances an agenda that connects NTD control to democracy, social justice, and pharmaceutical sovereignty. We argue that FSBEDIN offers practical lessons for implementing the World Health Assembly resolution on social participation and the WHO 2030 NTD roadmap. We propose policy measures for governments, the WHO, and development partners to institutionalize meaningful involvement of affected communities as a non-negotiable standard for NTD programs.

## Author summary

Neglected tropical diseases (NTDs) mainly affect people living in poverty, who are often discriminated against and far from decision-making spaces. Technical solutions (drugs, diagnostics, and guidelines) are insufficient if those most affected remain invisible in policy-making. In Brazil, people living with Chagas disease, leishmaniasis, leprosy, and other infections, together with community organizations, researchers, and health professionals, created the Brazilian Social Forum for Combating Infectious and Neglected Diseases (FSBEDIN). Over the past decade, the Forum has become a space where affected leaders learn, organize, and negotiate directly with governments and international partners. It has helped bring NTDs and their social consequences onto national agendas and supported the creation of a National Movement for Neglected Diseases. This Policy Platform shows how this experience can guide other countries. We argue that NTD programs should not be considered successful if affected communities are absent from decision-making. We outline practical steps for governments, the WHO, and donors to fund, protect, and measure meaningful participation as a core component of NTD policy.

## Policy summary points

- Neglected tropical diseases (NTDs) are a political problem as much as a biomedical one. In Brazil, the high burden of NTD is concentrated in areas where social rights, including the right to participate in public decision-making, are weakest.
- Participation is a core intervention for NTD, not an accessory. Brazil’s experience shows that organized, well-resourced participation of people affected can reshape national NTD policies and institutions.
- FSBEDIN is a social innovation in NTD governance. Over a decade, the Forum has linked diverse movements, produced annual political platforms, and expanded the presence of affected leaders in health councils, interministerial committees, and technical groups.
- Participation must be funded and institutionalized. Leadership schools, travel support, and communication networks were essential to sustaining the Forum and enabled the creation of a National Movement for Neglected Diseases in 2024.
- Global NTD targets should include explicit standards for social participation. Validation, certification, and external funding for NTD programs should require evidence of meaningful participation through participatory and deliberative governance mechanisms that involve affected communities throughout the policy cycle.
- South-South cooperation can help adapt this model. WHO, regional bodies, and donors should support networks of NTD social forums in high-burden countries, linking participation to human rights and pharmaceutical sovereignty agendas.

## NTD, inequity, and democratic deficits

More than one billion people worldwide live with or are at risk of neglected tropical diseases (NTD), which flourish where poverty, precarious housing, weak infrastructure, and limited access to health services persist [1,2]. In such settings, NTD are visible expressions of long-standing violations of social and human rights rather than isolated biomedical problems [3,4].

Brazil exemplifies this contradiction. Despite having a constitutionally guaranteed universal health system (*Sistema Único de Saúde*, SUS) and a long-standing legal and institutional architecture of social participation—including health councils and national health conferences, established by Federal Law No. 8,142/1990 and shaped by social movements—the country still accounts for a substantial share of NTD cases and deaths in the Americas [1,5,6]. Between 2016 and 2020, nearly 600,000 cases of ten selected NTDs were reported, and approximately 28.9 million people, about 14% of the population, were considered at risk, with the greatest burden falling on children, rural workers, and residents of historically marginalized regions in the North and Northeast [5,7].

At the same time, people affected by NTD have often remained politically invisible. Analyses of health-conference outputs and participatory spaces show that NTD-related conditions are frequently absent or marginal in national deliberations, despite their high burden [8]. When they are present, affected people are more often treated as targets of programs than recognized as political subjects. This invisibility is reinforced by stigma, structural racism, gender inequalities, environmental injustice, and an “expert-driven” model of decision-making that keeps communities at the periphery [3,4,9–12]. Across global health, however, the term “participation” encompasses a wide spectrum of arrangements, ranging from tokenistic consultation to deliberative co-governance that redistributes decision-making power; making participation meaningful, therefore, requires specifying who participates, with what authority, and through which accountability mechanisms [9–14].

In 2024, the 77th World Health Assembly formally acknowledged that this model is insufficient and adopted a resolution on “meaningful, inclusive, transparent and regular” social participation in health [13]. Implementing this resolution in the NTD field and aligning with the WHO roadmap and the Sustainable Development Goals (particularly SDG 3 and SDG 10) will require institutional arrangements that redistribute power, not just more consultations [1,13–15].

The Brazilian Social Forum for Combating Infectious and Neglected Diseases (FSBEDIN) emerged in this context as a social innovation in democratic health governance. Its trajectory offers concrete lessons on how to move from rhetoric about participation to enforceable standards in NTD policy.

## The Brazilian Social Forum: origins and design

FSBEDIN was established in 2016 amid a severe political and economic crisis characterized by an impeachment process, a long-term ceiling on social spending, and a broader rollback of social rights [16,17]. These developments threatened SUS financing and widened inequalities in access to care.

**Fig 1:**
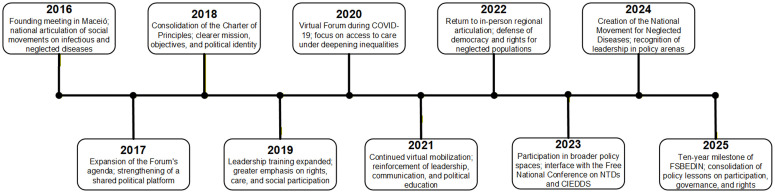
Timeline of key milestones in the Brazilian Social Forum for Combating Infectious and Neglected Diseases (FSBEDIN), 2016–2025. Source: Authors’ elaboration based on Forum letters, institutional records, and documentary analysis.

The timeline in Fig 1 summarizes the main political and organizational milestones in the Forum’s trajectory, including its founding in 2016, the consolidation of its Charter of Principles, the virtual meetings held during the COVID-19 pandemic, the expansion of leadership training activities, the creation of the National Movement for Neglected Diseases in 2024, and the 10-year milestone reached in 2025.

In this context, researchers, civil society organizations, and social movements convened a workshop in Rio de Janeiro on the interfaces between social movements and NGOs in the fight against neglected diseases. Participants included affected leaders and organizations working on Chagas disease, leishmaniases, leprosy, HIV/AIDS, and viral hepatitis, alongside universities, the Oswaldo Cruz Foundation, international NGOs, and global health initiatives. For the first time in Brazil, multiple disease-specific movements shared a space explicitly designed to articulate a common political agenda.

Three converging demands emerged from these debates. First, the defense of democracy and social rights as preconditions for tackling NTDs. Second, the defense and adequate financing of SUS, especially primary health care, were understood as central to guaranteeing access to diagnosis, treatment, and rehabilitation. Third, the recognition of affected people as political subjects with voice and voting power in health governance.

The 1st Brazilian Meeting of Social Movements Fighting Neglected Diseases, held in Maceió in 2016 alongside the Brazilian Society of Tropical Medicine congress, marked the formal creation of FSBEDIN. After preparatory meetings and early editions, the Forum consolidated a Charter of Principles, formally adopted in 2018, defining its mission as the defense of human and social rights to health within a democratic space of representation, support, empowerment, and articulation. Its strategic objectives were to protect the constitutional right to health, unite and expand the Forum, and increase the visibility of NTD and other infectious diseases [17].

Since then, FSBEDIN has organized annual meetings rotating across Brazilian regions (North, Northeast, Centre-West, and Southeast), including virtual editions during the COVID-19 pandemic (Table 1). Each meeting concludes with an open letter that synthesizes diagnoses, political analyses, and proposals directed to governments, international organizations, and society. Over time, these letters have evolved from a broad defense of SUS to more detailed agendas linking NTD to democracy, social protection, environmental justice, science and technology policy, and pharmaceutical sovereignty (Table 2) [5,17].

**Table 1:** Topics addressed during FSBEDIN meetings, by year and location, 2016–2025.

Year	Theme	Location
2016	Challenges, advances, and perspectives of social movements defending life, the Unified Health System (SUS), and fighting neglected diseases.	Maceió, Alagoas - Northeast region
2017	Defending the SUS and confronting infectious and neglected diseases.	Cuiabá, Mato Grosso – Central-West region
2018	Infectious and neglected diseases: silenced until when?	Recife, Pernambuco - Northeast region
2019	Access to health and inclusive development	Belo Horizonte, Minas Gerais - Southeast region
2020	The effects of the pandemic on access to healthcare for neglected tropical diseases (NTD) amid socioeconomic inequality. The role of leadership and social mobilization	Virtual - remote access
2021	Brazil: yesterday, today, and tomorrow for neglected populations. Confronting inequality and the social divide	Virtual - remote access
2022	In defense of democracy and rights for neglected populations.	Belém, Pará - North region
2023	Tomorrow will be another day: resuming social participation, reaffirming the SUS (Unified Health System), and ensuring equity in public policies.	Salvador, Bahia - Northeast region
2024	Advances and challenges in tackling NTD	São Paulo, São Paulo - Southeast region
2025	Ten Years of the Brazilian Social Forum for Combating Infectious Diseases: Advances and Challenges	João Pessoa, Paraíba - Northeast region

**Table 2:** Positions by thematic axis in FSBEDIN letters (2016–2025).

Thematic axis	Main demands	Letters (year)
Democracy and social justice	Defense of democracy; rule of law; tackling inequalities	All meetings (2016–2025)
Right to health and SUS	Valuing SUS: universality, comprehensiveness, and equity	All meetings (2016–2025)
SUS financing	Repeal of expenditure ceiling/austerity (Constitutional Amendment 95/2016); sustainable financing	Cuiabá (2017) to João Pessoa (2025)
Leadership and training	Popular education, leadership training, and empowerment	All meetings (2016–2025)
Invisibility and stigma	Combating prejudice; campaigns; symbolic milestones	Cuiabá (2017) to João Pessoa (2025)
Healthcare	Care pathways; strengthening PHC; priority groups	Recife (2018) to João Pessoa (2025)
Policies and intersectorality	Integrating health, social assistance, and education, tackling hunger	Recife (2018) to João Pessoa (2025)
Science, technology, and innovation	Strategic research; local production; compulsory licensing	All meetings (2016–2025)

The Forum also built internal structures for coordination, planning, communication, logistics, and culture, and strengthened alliances with universities, research institutes, international NGOs, and, more recently, the Ministry of Health. Participation diversified to include women, Black and Indigenous leaders, people with disabilities, favela residents, and rural workers [17]. This combination of political pluralism and territorial diversity became a source of legitimacy and influence [18].

## What has the Forum achieved—and what gaps remain?

Analysis of Forum letters (2016–2025), minutes, and associated documents reveals eight intertwined axes around which FSBEDIN organizes its political agenda [17]:

**Democracy and social justice.** Forum letters consistently denounce the erosion of democracy, the spread of misinformation, and authoritarian projects that undermine social rights. They link the high burden of NTD to broader patterns of democratic degradation and the commodification of health, echoing political science analyses of Brazil’s recent crisis [11,16,18,19].

**Right to health and SUS financing.** FSBEDIN calls for the repeal of constitutional austerity measures and for progressive, stable funding for SUS, with emphasis on primary health care and community-based approaches to NTD. These positions resonate with long-standing debates about the tension between universality and underfunding in SUS [14].

**Leadership and training.** One of the Forum’s most distinctive contributions is its investment in political education for people affected. Three national leadership courses (2019, 2021, and 2024) combined technical content on NTD with modules on human rights, public policy, communication, negotiation, and advocacy. These processes culminated in 2024 in the formal creation of a National Movement for Neglected Diseases, designed and led by affected people themselves [17].

**Invisibility, stigma, and care.** Forum documents denounce stigma, discrimination, and structural racism, and call for integrated lines of care that connect surveillance, diagnosis, treatment, rehabilitation, and social protection, especially through primary health care. They demand attention to mental health and disability and insist that NTD care must be guided by lived experience, not only by epidemiological indicators [3–8,17].

**Science, technology, innovation, and sovereignty.** FSBEDIN links NTD control to a broader agenda of pharmaceutical and technological sovereignty. It advocates public production of medicines, diagnostics, and vaccines; supports Productive Development Partnerships; and defends measures such as compulsory licensing when intellectual property regimes obstruct access [20–25]. This aligns with analyses of imbalances between disease burden, research funding, and technology development for NTD [20–23].

**Fig 2:**
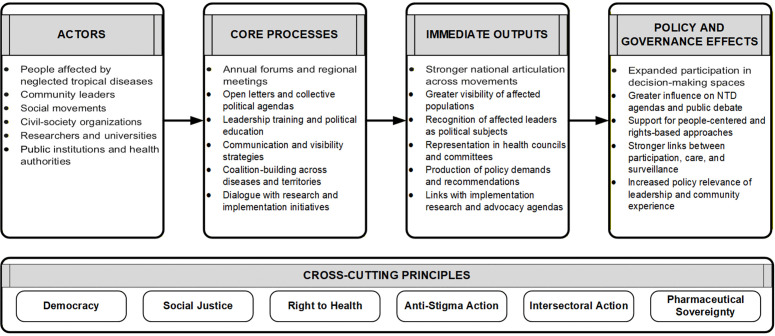
FSBEDIN as a platform for participation, leadership, and policy influence in the field of neglected tropical diseases in Brazil. Source: Authors’ elaboration based on Forum letters, institutional records, and documentary analysis.

Beyond discourse, the Forum has contributed to concrete institutional changes (Fig 2): increased presence of NTD leaders in local, state and national health councils; participation in the organization of Brazil’s first Free National Conference on NTD (2023); involvement in implementation research initiatives developed in partnership with research institutions and national and local governments, such as IntegraChagas Brasil and CUIDA Chagas; representation in the Interministerial Committee for the Elimination of Tuberculosis and other Socially Determined Diseases (CIEDDS), created in 2023 [26]; the approval of Law No. 14,977/2024, which assigns public pharmaceutical laboratories a strategic role in the production of active ingredients for socially determined diseases; and recognition as a civil-society counterpart in the interministerial program *Brasil Saudável - Unir para Cuidar*, which coordinates 13 ministries around socially determined diseases, including NTD [25].

The diagram in Fig 2 illustrates how FSBEDIN connects affected communities, social movements, researchers, civil-society organizations, and public institutions through leadership training, annual forums, open letters, advocacy, and coalition-building. These processes contribute to greater visibility of affected populations, stronger participation in governance spaces, implementation research partnerships, and broader policy influence in neglected tropical disease agendas.

**Important gaps remain.** Participation remains uneven across states and municipalities; many local programs lack structured mechanisms to involve affected communities in planning, budgeting, or evaluation. Funding for social participation and community organizations is precarious, often dependent on short-term projects and personal sacrifice. NTD agendas also remain low-profile in broader development and climate debates, despite clear intersections with labor conditions, environmental change, and food insecurity [1,2,5,7,22]. These gaps point to structural problems in the way national and global NTD policies have historically treated participation as desirable yet optional and often symbolic.

**Reflexivity and limitations.** Several authors have long-standing engagement with NTD policy and have participated in FSBEDIN-related processes as collaborators, researchers, or institutional partners. To mitigate potential bias, we triangulated multiple sources, including letters, minutes, and institutional records, and employed a transparent documentary analysis approach. However, this Policy Platform was produced within conventional academic authorship rules and does not fully resolve the tension between academic representation and the broader protagonism of affected communities. It is not an ethnography and does not claim to represent all local experiences across Brazil; further research should assess participation processes and outcomes in specific policy arenas.

## Policy implications: from experience to standards

Drawing on FSBEDIN’s trajectory and on theoretical and empirical literature on participation, social movements, and NTD, we propose five sets of policy measures for governments, the WHO, and development partners.

### Embed affected communities in formal decision-making

- Reserve voting seats for representatives of people affected by NTD in national committees, health councils, and interministerial bodies that address socially determined diseases.
- Agree on transparent and plural selection processes led by civil-society platforms, rather than appointments decided solely by governments.
- Guarantee stipends, travel, and accessibility support (including childcare and disability-related support) so participation does not depend on personal resources.

This shifts participation from consultation to co-governance, consistent with democratic theories that emphasize shared decision-making and “deep democracy” in health [9–11,14].

### Create sustainable funding lines for social participation

- Establish national and subnational budget lines dedicated to community-based organizations, leadership schools, and communication initiatives on NTD.
- Encourage donors and philanthropies to allocate a minimum proportion of NTD investments to civil society and community-led activities, including core support.
- Include affected organizations as direct recipients of funds, not only as implementing partners under technical projects.

Without stable funding, forums such as FSBEDIN rely on intermittent grants and unpaid labor, limiting their reach and increasing burnout among leaders [17].

### Make meaningful participation a criterion for NTD program success

- Incorporate indicators on participation of affected communities into WHO NTD monitoring frameworks and national scorecards.
- Require participatory and deliberative forums involving affected communities as part of verification and validation processes for NTD elimination or control, alongside epidemiological and entomological indicators.
- Co-develop participatory evaluation tools with social movements, allowing communities to assess programs using their own priorities as well as official targets.

This aligns NTD policy with SDG commitments to inclusive institutions and accountability [11,14,15].

### Co-produce information and communication

- Support community-led media, local bulletins, and digital platforms that share accessible information on NTD, rights, and services in multiple languages and formats.
- Integrate community-generated data on barriers to diagnosis, treatment interruptions, stock-outs, or discrimination into official surveillance and planning.
- Partner with schools, universities, and public broadcasters to combat stigma and promote a rights-based narrative of NTD, emphasizing affected people as leaders rather than victims.

FSBEDIN’s experience shows that communication is not just dissemination; it is a tool for building collective identity and political capability [17].

### Foster South-South cooperation among NTD social forums

- Encourage WHO regional offices, regional NTD networks, and donors to support exchanges between Brazilian movements and counterparts in other high-burden countries.
- Create regional forums of people affected by NTD linked to existing technical and policy platforms.
- Use multilateral forums and global spaces (such as G20, BRICS, and UN high-level meetings) to highlight organized civil society as part of broader debates on health, climate justice, and pharmaceutical sovereignty [20–24,26].

Such cooperation can help adapt the Brazilian model to different political systems while recognizing that no model is exportable without local reinvention. [12].

Adopting and adapting the policy measures proposed here, embedding affected communities in governance, funding participation, making it an explicit criterion for program success, co-producing information, and fostering South–South cooperation, would be a concrete step toward aligning NTD agendas with human-rights principles and the World Health Assembly resolution on social participation [3,4,13,14].

## Conclusion: participation as a nonnegotiable standard for NTD policy

Over the past decade, FSBEDIN has moved from a crisis-driven experiment to a recognized actor in national health governance [17]. By creating a stable platform where affected people organize, learn, and negotiate, FSBEDIN has helped transform NTD from a silent expression of neglect into a field of explicit political dispute over rights, resources, and knowledge.

The core message for global NTD policy is straightforward but demanding: no country will meet the 2030 NTD targets or the SDG promise to “leave no one behind” if people affected remain excluded from decision-making processes [1,3,15]. Technical interventions cannot compensate for democratic deficits.

We therefore argue that meaningful participation of affected communities should be treated as a nonnegotiable standard for NTD programs, alongside safety, efficacy, and cost-effectiveness of interventions.

Brazil’s experience shows that when people affected have organized platforms, resources, and institutional access, they do not merely “give testimony”; they change the terms of the debate. Recognizing and resourcing this political role is essential if NTD policy is to be worthy of its name.

## References

[1] World Health Organization. Global report on neglected tropical diseases 2024: executive summary. Geneva: WHO; 2024. Available from: https://iris.who.int/bitstream/handle/10665/376808/B09040-eng.pdf

[2] Feng L, Mo G, Liu Z, Liu Q. Global, regional, national burden, trends and health inequality of neglected tropical diseases and malaria from 1990 to 2021. BMC Infect Dis. 2025;25(1):1649. doi: 10.1186/s12879-025-12068-x 41291509 PMC12649024

[3] Sun N, Amon JJ. Addressing inequity: neglected tropical diseases and human rights. Health Hum Rights. 2018;20(1):11–25. 30008549 PMC6039727

[4] Hunt P. Neglected diseases: a human rights analysis. Geneva: World Health Organization; 2007. Available from: https://iris.who.int/bitstreams/b0a9c710-6b9d-445a-88d0-d596258e345e/download

[5] Brasil. Doenças tropicais negligenciadas no Brasil: morbimortalidade e resposta nacional no contexto dos ODS. Brasília: Ministério da Saúde; 2024. Available from: https://www.gov.br/saude/pt-br/centrais-de-conteudo/publicacoes/boletins/epidemiologicos/especiais/2024/boletim-epidemiologico-de-doencas-tropicais-negligenciadas-numero-especial-jan-2024/@@download/file

[6] Martins-Melo FR, Ramos AN Jr, Alencar CH, Heukelbach J. Trends and spatial patterns of mortality related to neglected tropical diseases in Brazil. Parasite Epidemiol Control. 2016;1(2):56–65. doi: 10.1016/j.parepi.2016.03.002 29988194 PMC5991825

[7] Brasil. Doenças tropicais negligenciadas no Brasil: impacto na morbimortalidade das crianças (2010–2023). Brasília: Ministério da Saúde; 2025. Available from: https://www.gov.br/saude/pt-br/centrais-de-conteudo/publicacoes/boletins/epidemiologicos/especiais/2025/boletim-epidemiologico-de-doencas-tropicais-negligenciadas-numero-especial-jan-2025.pdf

[8] Pegaiani KNA, Pinto NS, Batiston AP, Santos MLM, Cabral KV, Belloc MM, et al. Health and leprosy conferences: sayings and silences about the neglected disease and its stigmas. Saude Soc. 2023;32(3):e210889. doi: 10.1590/S0104-12902023210889en

[9] Pateman C. Participation and democratic theory. Cambridge: Cambridge University Press; 1970. Available from: https://www.cambridge.org/core/books/participation-and-democratic-theory/75E1EDCA6842303901349FB5D3B0F261

[10] Borba J. Participação política: uma revisão dos modelos de classificação. Soc Estado. 2012;27(2):263–88. doi: 10.1590/s0102-69922012000200004

[11] Miguel LF. Resgatar a participação: democracia participativa e representação política no debate contemporâneo. Lua Nova. 2017;(100):83–118. Available from: https://www.scielo.br/j/ln/a/dLkRQT88JKty5dWBWKKm4vL/abstract/?lang=pt

[12] Scherer-Warren I. Das mobilizações às redes de movimentos sociais. Soc Estado. 2006;21(1):109–30. doi: 10.1590/s0102-69922006000100007

[13] World Health Organization. World Health Assembly endorses resolution on social participation. Geneva: WHO; 2024. Available from: https://www.who.int/news/item/29-05-2024-world-health-assembly-endorses-resolution-on-social-participation

[14] Palmquist B. Equity, participation, and power: achieving health justice through deep democracy. J Law Med Ethics. 2020;48(3):393–410. doi: 10.1177/1073110520958863 33021188

[15] United Nations Brazil. Objetivos de Desenvolvimento Sustentável — ODS 3. Brasília: ONU Brasil; 2024. Available from: https://brasil.un.org/pt-br/sdgs/3

[16] Avritzer L. Bolsonarismo: movimento ou forma de governo. In: Avritzer L, Kerche F, Marona M, editors. Governo Bolsonaro: retrocesso democrático e degradação política. São Paulo: Autêntica; 2021. Available from: https://bibliotecadigital.tse.jus.br/server/api/core/bitstreams/ac2c69f4-face-492d-9f11-3c6ee4227fb3/content

[17] Ramos AN Jr, Araújo CEL, Souza EA, Cunha ESM, Silva JAM, Pinheiro ES, et al. Fórum Social Brasileiro de Enfrentamento das Doenças Infecciosas e Negligenciadas (FSBEDIN). Documento síntese de história: Brasil, 2025. Fortaleza: Universidade Federal do Ceará; 2025. Available from: http://www.repositorio.ufc.br/handle/riufc/83259

[18] Santos BS, Avritzer L. Introdução: para ampliar o cânone democrático. In: Santos BS, editor. Democratizar a democracia: os caminhos da democracia participativa. Rio de Janeiro: Civilização Brasileira; 2004. p. 39–82.

[19] Gohn MG. Movimentos sociais na contemporaneidade. Rev Bras Educ. 2011;16(47):333–61. doi: 10.1590/S1413-24782011000200005

[20] Astone DP. Scarcity, property rights, irresponsibility: how intellectual property deals with neglected tropical diseases. Law Crit. 2024;34(1):145–64. doi: 10.1007/s10978-022-09324-3 37521680 PMC9244109

[21] Mahoney RT, Morel CM. A global health innovation system (GHIS). Health Res Policy Syst. 2006;4:2. doi: 10.1186/1478-4505-4-2 16460571 PMC1379643

[22] Fonseca B de P, Albuquerque PC, Zicker F. Neglected tropical diseases in Brazil: lack of correlation between disease burden, research funding and output. Trop Med Int Health. 2020;25(11):1373–84. doi: 10.1111/tmi.13478 32860446

[23] Beck EJ, Mandalia S, Dongmo Nguimfack B, Pinheiro E, ’t Hoen E, Boulet P, et al. Does the political will exist to bring quality-assured and affordable drugs to low- and middle-income countries? Glob Health Action. 2019;12(1):1586317. doi: 10.1080/16549716.2019.1586317 30983547 PMC6484498

[24] Lopes LMN, Andrade EIG, Borde EMS. The end of patent extensions and the productive development partnerships: effects on access to medicines in Brazil. Saude Soc. 2024;33(1):e220461. doi: 10.1590/S0104-12902024220461en

[25] Brasil. Estratégia para o Complexo Econômico-Industrial da Saúde (CEIS): anúncio de investimento até 2026. Brasília: Ministério do Desenvolvimento, Indústria, Comércio e Serviços; 2023. Available from: https://www.gov.br/mdic/pt-br/assuntos/noticias/2023/setembro/governo-lanca-estrategia-para-desenvolver-complexo-economico-industrial-da-saude-com-investimento-de-r-42-bilhoes-ate-2026

[26] Brasil. Decreto nº 11.494, de 17 de abril de 2023 — institui o Comitê Interministerial para a Eliminação da Tuberculose e outras Doenças Determinadas Socialmente (CIEDDS). Brasília: Presidência da República; 2023. Available from: https://www.planalto.gov.br/ccivil_03/_ato2023-2026/2023/decreto/D11494.htm

